# hsa-miR-3177-5p and hsa-miR-3178 Inhibit 5-HT1A Expression by Binding the 3′-UTR Region *in vitro*

**DOI:** 10.3389/fnmol.2019.00013

**Published:** 2019-01-31

**Authors:** Xue Wu, Mei Ding, Yi Liu, Xi Xia, Feng-ling Xu, Jun Yao, Bao-jie Wang

**Affiliations:** School of Forensic Medicine, China Medical University, Shenyang, China

**Keywords:** HTR1A gene, 5-HT1A receptor, hsa-miR-3177-5p, hsa-miR-3178, neuropsychiatric disorders

## Abstract

Abnormal expression of the 5-HT1A receptor, which is encoded by the HTR1A gene, leads to susceptibilities to neuropsychiatric disorders such as depression, anxiety, and schizophrenia. miRNAs regulate gene expression by recognizing the 3′-UTR region of mRNA. This study evaluated the miRNAs that might identify and subsequently determine the regulatory mechanism of HTR1A gene. Using the HEK-293, U87, SK-N-SH and SH-SY5Y cell lines, we determined the functional sequence of the 3′-UTR region of the HTR1A gene and predicted miRNA binding. Dual luciferase reporter assay and Western Blot were used to confirm the effect of miRNA mimics and inhibitors on endogenous 5-HT1A receptors. In all cell lines, gene expression of the −17 bp to +443 bp fragment containing the complete sequence of the 3′-UTR region was significantly decreased, although mRNA quantification was not different. The +375 bp to +443 bp sequence, which exhibited the most significant change in relative chemiluminescence intensity, was recognized by hsa-miR-3177-5p and hsa-miR-3178. In HEK-293 and U87 cells, hsa-miR-3177-5p significantly inhibited the 5-HT1A receptor expression, while a hsa-miR-3178 inhibitor up-regulated HTR1A gene expression in SK-N-SH and SH-SY5Y cells. By constructing the pmirGLO-vector with the mutated HTR1A gene, we further confirmed that hsa-miR-3177-5p recognized the HTR1A gene tgtacaca at +377 bp to +384 bp, and the +392 bp to +399 bp fragment cgcgccca was identified by hsa-miR-3178. hsa-miR-3177-5p and hsa-miR-3178 had significant inhibitory effects on expression of the HTR1A gene and 5-HT1A receptor and may directly participate in the development of neuropsychiatric diseases.

## Introduction

The serotonin nervous system is associated with the pathological mechanisms in various neuropsychiatric disorders, such as depression (Lemonde et al., 2003; Dorado et al., 2007), anxiety (Heisler et al., 1998; David et al., 2005) and schizophrenia (Gu et al., 2013; Lin et al., 2015; Takekita et al., 2016). 5-HT neurons originate in the raphe nuclei of the brain and regulate emotions and mood by dominating 5-HT1A receptor-rich regions, including the cortex, hippocampus, diaphragm, and amygdala (Törk, 1990; Ou et al., 2000; Lemonde et al., 2004). Related animal and autopsy studies have confirmed that a reduction in 5-HT1A postsynaptic receptors and over-expression of 5-HT1A presynaptic receptors (auto-receptors), which decrease the activity and content of serotonin neurotransmitters at an overall level, are present in patients with major depression (Arango et al., 2001; Miller et al., 2009; Parsey et al., 2010). However, evidence has also suggested that the number of 5-HT1A auto-receptors in depressive suicidal patients was reduced by 43% (Arango et al., 2001). For cognitive function in patients with schizophrenia, the dopamine neurotransmitter in the prefrontal cortex plays a crucial role, and the HTR1A gene interacts with the COMT gene to regulate the function of the dopamine system (Bosia et al., 2014). In addition, autopsy studies have determined that there is indeed an increase in 5-HT1A receptors in the prefrontal cortex of patients with schizophrenia (Burnet et al., 1996; Sumiyoshi et al., 1996). Therefore, irregular expression of the 5-HT1A receptor may be an important factor causing susceptibility to developing neurological diseases.

The 5′ flanking region of the HTR1A gene is highly conserved in the humans, mice and rats. About seven transcription factors have been shown to have important regulatory effects on 5-HT1A receptor expression (Albert and Fiori, 2014). In the initial 715 bp of the HTR1A gene (ATG + 1), the sequence contains multiple conserved MAZ I-IV and Sp1 recognizing sites, which drive basal HTR1A gene expression (Parks and Shenk, 1996; Meijer et al., 2000). Moreover, a glucocorticoid response element (nGRE) and a silencing transcript region, which contains a RE-1 element and two tandem DRE elements, significantly inhibit the expression of the 5-HT1A receptor (Ou et al., 2001; Lemonde et al., 2004; Albert and Fiori, 2014).

Another important regulatory domain of the gene—the functional sequences located at the 3′-UTR or 3′ flanking region- is poorly understood. The 3′-UTR sequence blocks initiation of translation or increases mRNA degradation by binding miRNAs (Chak et al., 2016), which ultimately leads to decreased expression of the target protein (Bartel, 2004). The miRNA “seed” region spans 2–7 bases at the 5′ end, and more than 60% of human encoded protein genes are recognized by at least one conserved miRNA (Ha and Kim, 2014). More importantly, abnormal miRNA regulation is involved in not only the occurrence of human tumors (Lujambio and Lowe, 2012), but also disorders in nerve development (Im and Kenny, 2012). The dual luciferase reporter assay demonstrated that the miR-135 strongly inhibited HTR1A and SLC6A4 genes, resulting in a 30%–50% decrease in transcriptional activity. MiR-135 expression was also found to be significantly reduced in the brain and blood of depressed patients. In addition, the 3′-UTR sequence of the HTR1A gene was also combined with miR-335, miR-181c and miR-26a (Issler et al., 2014). The researchers also found that there was a reduction of the variable splicing with brain-domain dependence in patients with major depression. The alternative splicing in the human HTR1A gene 3′-UTR region stabilized the HTR1A gene RNA by antagonizing the inhibitory effect of miR-135 on the HTR1A gene expression (Le François et al., 2018). Related study has shown that the inhibitory effect of miR-96 on the HTR1B gene is attenuated by the G allele of the polymorphism rs13212041, while the mice with reduced HTR1B gene expression exhibited the more prominent aggressive behavior (Jensen et al., 2009). The SLC6A4 gene, which is involved in the susceptibility of several psychiatric diseases, was negatively regulated by the miR-15 and miR-16 in humans and rats (Moya et al., 2013). Moreover, the decrease in the SLC6A4 mRNA expression might also be due to the changes in certain SNPs that disrupted the binding of miR-545 and miR-590-3p (Iurescia et al., 2017). Dopamine system that interacts with the serotonin nervous system is also regulated by the miRNAs. The expression of DRD2 mRNA and protein was reduced by the over-expressed miR-9 and miR-326. Perhaps the DRD2 receptor participants in the development of schizophrenia and other psychiatric diseases, making the functional sites of miRNAs be the potential targets for the treatment of schizophrenia (Shi et al., 2014). Additionally, the abnormal regulation of the miR-9, miR-29, miR-137 and miR-106 was found in the prefrontal cortex of patients with schizophrenia (Camkurt et al., 2016; Curtis and Emmett, 2017). The expression of miR-106 was also observed to be in a diametrically opposite trend in the plasma of patients with major depression and ADHD (Kandemir et al., 2014; Liu et al., 2014). The above evidences fully demonstrated that the miRNAs were involved in the pathological mechanisms of the neuropsychiatric disorders.

By searching for functional fragments of the 3′-UTR region in the HTR1A gene, we predicted possible miRNA binding and performed miRNA mimic and inhibitor experiments *in vitro* to explore the effects of miRNAs on the expression of 5-HT1A receptor.

## Materials and Methods

### Construction of the pmirGLO-HTR1A Recombinant Vector

PCR amplification of the target fragments was performed with primers (Table 1) that were introduced to Nhe and Xho restriction endonuclease sites at the 5′ end. Purified PCR products were then cloned into the pGM-T vectors. Transformation of the recombinant vectors utilized T-fast competent cells. Finally, the correct target fragments were screened by the Sanger sequencing and cloned into pmirGLO vectors. The sequence ranging from −17 bp to +1,066 bp in the HTR1A gene 3′-UTR region (the next base of the stop codon being +1) was the longest fragment and was synthesized as an amplification template for other sequences as follows: −17 bp to +443 bp, −17 bp to +374 bp, −17 bp to +326 bp, −17 bp to +241 bp and −17 bp to +99 bp. All recombinant vectors were used in subsequent eukaryotic cell experiments.

**Table 1:** PCR primer sequences.

Primer name	Sequence
HTR1A-3′UTR (−17 to +1066) F	5′-CTAGCTAGCTAGAGTGATGACGGAG-3′
HTR1A-3′UTR (−17 to +1066) R	5′-CCGCTCGAGTCCTGTAAGTCAG-3′
HTR1A-3′UTR (−17 to +443) R	5′-CCGCTCGAGTTCTAGAAGTTGG-3′
HTR1A-3′UTR (−17 to +374) R	5′-CCGCTCGAGTTTTGGATTTTCT-3′
HTR1A-3′UTR (−17 to +326) R	5′-CCGCTCGAGTTTAGGAGATAGAA-3′
HTR1A-3′UTR (−17 to +241) R	5′-CCGCTCGAGAACTCTCTGAATTT-3′
HTR1A-3′UTR (−17 to +99) R	5′-CCGCTCGAGTTATCTTAAGTGTT-3′

*Primers for PCR amplification of target fragments in the HTR1A gene 3′-UTR region. The numbers represent the start and end positions of the amplified fragments of the HTR1A gene (the next base of the stop codon is +1)*.

### Cell Culture

Transfection of recombinant vectors was conducted with four cell lines: HEK-293, U87, SK-N-SH and SH-SY5Y. HEK-293 and U87 cells were cultured in HyClone^®^ DMEM high glucose medium containing 10% fetal bovine serum (Thermo Fisher Scientific, Chelmsford, MA, USA), and the SK-N-SH cell line was treated with KeyGEN BioTECH ^®^ DMEM high glucose medium with 0.110 g/L sodium pyruvate containing 15% fetal bovine serum. SH-SY5Y cells were cultured in Corning^®^ DMEM/F12 1:1 mix medium +15% fetal bovine serum. All cells were cultured to a density of more than 90% at 37°C in 5% CO_2_ + 95% mixed air.

### miRNA Prediction

MiRNA prediction of the functional sequence in the HTR1A gene 3′-UTR region was performed using the TargetScanHuman 7.2^1^, miRbase^2^ and RNAhybird.

### Transient Transfection and Dual Luciferase Reporter Assay

#### Transient Transfection of pmirGLO-HTR1A Recombinant Vectors or/and siRNA of the Dicer

Cells with a density of 90% were inoculated into 24-well plates at 2 × 10^5^ cells per well and cultured for 36–48 h. The eukaryotic cells were transfected with 2 μL Lipofectamine^®^ 3,000 reagent (Invitrogen, Carlsbad, CA, USA) + 1 μg pmirGLO-HTR1A recombinant plasmid per well for 24 h. To knock-down the endogenous Dicer enzyme and validate the Dicer-mediated miRNA regulation, we transfected 1 μL siRNA-1660 (20 pmol) + 200 ng pmirGLO vectors per well into the four cell lines (siRNA-1660: 5′-GCACCCAUCUCUAAUUAUATT-3′). Cell lysates or total RNAs were collected for the dual luciferase reporter assay and real-time PCR. Each sample was tested in triplicate per experiment with a total of three experiments.

#### Co-transfection of pmirGLO-HTR1A (−17–+443) With the hsa-miR-3177-5p or hsa-miR-3178

When the cells were in exponential growth, 2 μL Lipofectamine^®^ 2,000 reagent per well was incubated with 100 ng pmirGLO-HTR1A (−17–+443)/100 ng pmirGLO-HTR1A (−17–+443) mutation + 1 μL (0.264 μg) hsa-miR-3177-5p or hsa-miR-3178 mimic/inhibitor or miRNA NC mimic/inhibitor (GenePharma, Shanghai, China). After transfection for 24 h, the cells were washed with PBS and the cell lysates were collected for measurement of firefly and renilla luciferase.

#### Independent Transfection of hsa-miR-3177-5p or hsa-miR-3178 Mimic/Inhibitor or miRNA NC Mimic/Inhibitor

Cells with a density of 90% were seeded in 6-well plates at 1 × 10^6^ cells/well. Four cell lines were transfected with 8 μL Lipofectamine^®^ 2,000 reagent + 5 μL (1.3 μg) hsa-miR-3177-5p or hsa-miR-3178 mimic/inhibitor or miRNA NC mimic/inhibitor. Proteins and total RNAs from SK-N-SH, HEK-293, U87 and SH-SY5Y were extracted at 48 or 72 h after transfection. Endogenous 5-HT1A receptor expression was detected using Western Blot and real-time PCR experiments.

### Dual Luciferase Reporter Assay

Cells in each well were lysed with 100 μL 1× PLB. A total of 20 μL luciferase substrate and 1× STOP reagent were added to 20 μL cell lysates to detect firefly luciferase (LUC) and renilla luciferase (TK) protein expression (Promega). LUC/TK is the relative chemiluminescence intensity.

### Real-Time PCR

Total RNAs from the transfected cells were extracted according to the following procedures. A total of 400 μL or 800 μL Trizol were added to 24-well or 6-well plates, and the cell lysates were successively treated with chloroform, isopropanol and 75% ethanol. Total RNAs were then dissolved in 15 μL DEPC water for several hours and quantified using a UV spectrophotometer. No more than 1 μg RNA was included in each 20 μL cDNA reaction system. Real-time PCR amplification of cDNA utilized the following three pairs of primers: pmirGLO-LUC F: 5′-TACACCTTCGTGACTTCCCATTT-3′ R: 5′-ATGACTGAATCGGACACAAGCG-3′; pmirGLO-RLUC (TK) F: 5′-GGGAAGGGACTGGCTGCTAT-3′ R: 5′-TGCTCTTCGTCCAGATCATCCT-3′; HTR1A F: 5′-GACTACGTGAACAAGAGGACGCCC-3′ R: 5′-ATGAGCAGCAGCGGGATGTAGAAAG-3′. Real-time PCR was performed using the SYBR dye method (Takara, Dalian, China).

### Western Blot

Total proteins from the four transfected cell lines were extracted by NP-40 and PMSF. Quantified protein samples were separated by electrophoresis in 10% denatured polypropylene gel and transferred using PVDF membrane for 1 h at 100 V. After blocking with 8% skimmed milk, primary antibodies for 5-HT1A (Thermo Scientific™) and beta-actin (Abbkine, USA) were diluted with TBS-T at a ratio of 1:1,000 and 1:2,000, respectively. Membranes were incubated overnight at 4. Diluted secondary antibody (Abbkine, USA, 1:5,000) was incubated with the membrane for 2 h, which had been washed three times with TBS-T. Target protein expression was detected by ECL luminescent solution and the Tanon-5500 chemiluminescence imaging analysis system. Detection of the target protein from the same sample was repeated three times. The 5-HT1A receptor and the internal reference beta-actin are 35–55 KD and 42 KD, respectively.

### Statistical Analysis

The relative chemiluminescence intensity LUC/TK from the same sample was expressed as the mean ± standard deviation. One-way analysis of variance (ANOVA) was used to compare the differences among multiple samples. However, comparison between the two samples was performed using the LSD-*T* test. Real-time PCR was calculated by the 2^−ΔΔCT^ method to compare differences in mRNA expression. Quantification of protein expression from Western Blot (gray values) were determined using ImageJ software and differences in protein expression were compared by Student’s *t*-test. *P* < 0.05 represents a significant statistical difference. Statistical calculations were performed with SPSS 20.0 software.

## Results

### The Relative Chemiluminescence Intensities of pmirGLO-Basic, pmirGLO-HTR1A (−17–+443) and pmirGLO-HTR1A (−17–+374) Were Significantly Different

**Figure 1:**
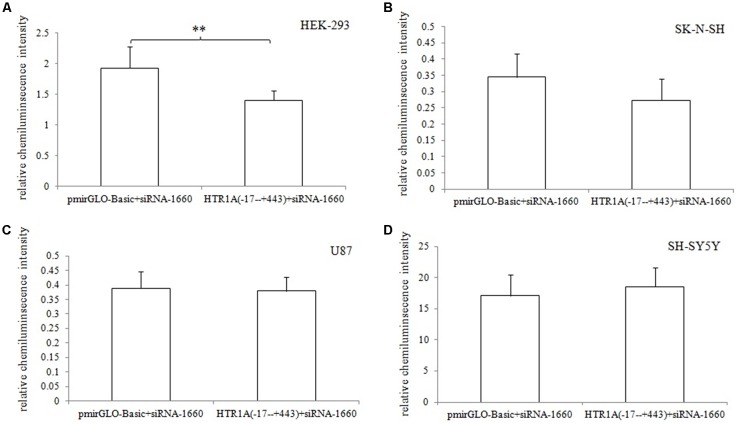
Effect of the Dicer knock-down on the inhibitory function of 3′-UTR sequence **(A–D)**. When the endogenous Dicer enzyme of the four cell lines were knocked down, we found that the inhibition of the 3′-UTR sequence was not significant in SK-N-SH and U87 cells. The results indicated that the down-regulation of gene expression by the 3′-UTR sequence might be exerted by the Dicer-mediated miRNAs. **0.001 ≤ *p* < 0.02.

**Figure 2:**
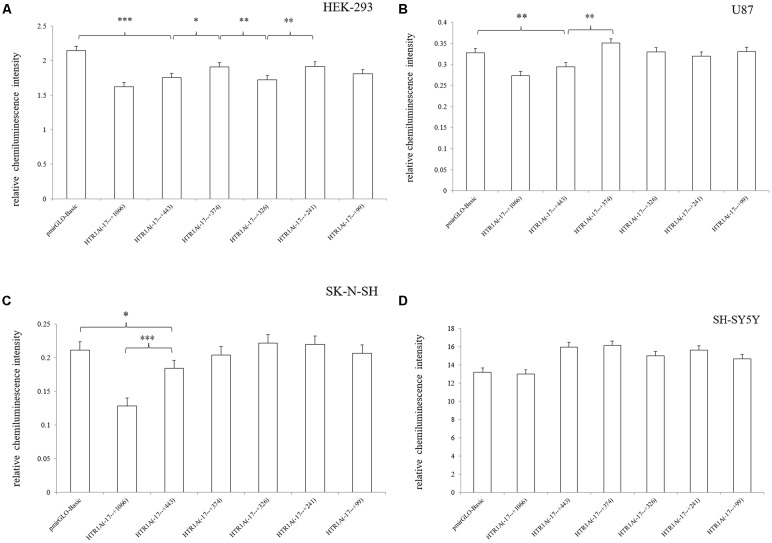
Relative chemiluminescence intensities of the functional sequences of the HTR1A gene 3′-UTR region **(A–D)**. In HEK-293, U87 and SK-N-SH cell lines, the relative chemiluminescence intensity of the 3′-UTR complete sequence ranging from −17 bp to +443 bp was significantly decreased. The sequence +375 bp to +443 bp also showed the strongest inhibitory effect on protein expression in HEK-293 and U87 cells. Relative chemiluminescence intensity of each sample is expressed as the mean ± SD, and the LSD-T test was used to compare the two samples. *0.02 ≤ *p* < 0.05, **0.001 ≤ *p* < 0.02, ***p < 0.001.

In HEK-293, U87 and SK-N-SH cells, the complete 3′-UTR sequence of the HTR1A gene from −17 bp to +443 bp showed a significant decrease in relative chemiluminescence intensity compared with pmirGLO-Basic (*p* < 0.001, *p* = 0.006 and 0.02, respectively). However, when the endogenous Dicer enzyme was knocked down, the inhibitory function of the −17 bp to +443 bp sequence was apparently disappeared in SK-N-SH and U87 cell lines (Figure 1). In addition, when comparing the target fragments −17 bp to +443 bp and −17 bp to +374 bp, protein expression also exhibited significant statistical differences in the HEK-293 and U87 cell lines (*p* = 0.035 and *p* < 0.001). Relative chemiluminescence intensities of −17 bp to +374 bp vs. −17 bp to +326 bp and −17 bp to +326 bp vs. −17 bp to +241 bp were only significant in the HEK-293 cell lines (*p* = 0.012 and 0.009; Figure 2).

### No Differences in mRNA Expression Among Target Fragments of the HTR1A gene 3′-UTR Region

**Figure 3:**
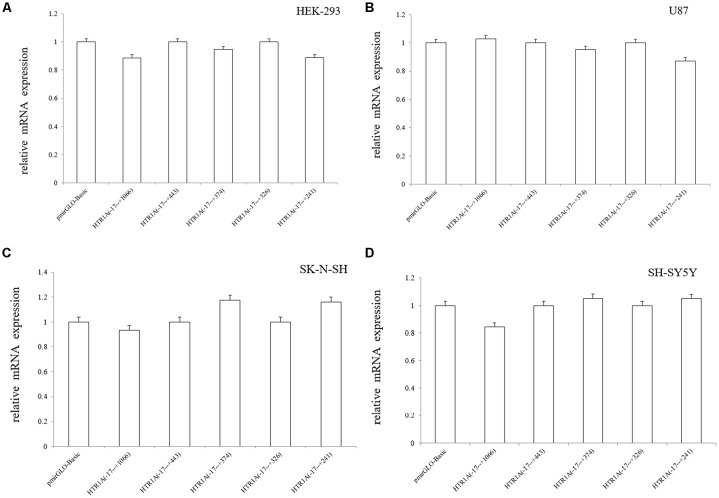
Relative mRNA expression of the functional sequences of the HTR1A gene 3′-UTR region **(A–D)**. Relative mRNA expression of 3′-UTR functional sequences in the HTR1A gene was not significantly different in the four cell lines. Real-time PCR was calculated by the 2^−ΔΔCT^ method, and the LSD-T test was used to compare the two samples.

In the four cell lines, real-time PCR experiments confirmed that mRNA expression of pmirGLO-Basic vs. −17 bp to +1066 bp, −17 bp to +443 bp vs. −17 bp to +374 bp and −17 bp to +326 bp vs. −17 bp to +241 bp did not show statistically significant differences (*p* > 0.05; Figure 3).

### hsa-miR-3177-5p and hsa-miR-3178 Might Recognize the Target Fragment +375 bp to +443 bp

**Figure 4:**
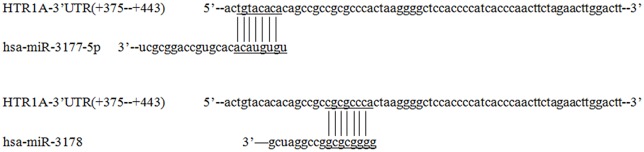
Prediction of miRNAs that recognize the HTR1A gene 3′-UTR region. Based on the number of combined bases and cells expression, TargetScanHuman 7.2 software showed that hsa-miR-3177-5p and hsa-miR-3178 were most likely to regulate expression of the HTR1A gene and the recognition sequences located at +377 bp to +384 bp and +392 bp –+399 bp. The numbers represent the position of the gene (the next base of the stop codon is +1) and the horizontal and vertical lines represent matching bases.

TargetScanHuman 7.2, miRbase and RNAhybird was used to predict potential miRNA recognition of the sequence +375 bp to +443 bp. According to the number of matched bases and miRNA expression in four cell lines (miRNA real-time PCR was used to detect endogenous miRNA expression in cells; Supplementary Figure S1), it was confirmed that hsa-miR-3177-5p bound to tgtacaca of the +377 bp to +384 bp and hsa-miR-3178 recognized cgcgccca located at +392 bp to +399 bp (Figure 4).

### hsa-miR-3177-5p and hsa-miR-3178 Significantly Down-Regulated the Relative Chemiluminescence Intensity of the −17 bp to +443 bp Sequence

After co-transfected of pmirGLO-HTR1A (−17–+443) with hsa-miR-3177-5p mimic/inhibitor or miRNA NC mimic/inhibitor, we found that the relative chemiluminescence intensity of the pmirGLO-HTR1A (−17–+443) + hsa-miR-3177-5p mimic was lower than that of the pmirGLO-HTR1A (−17–+443) + miRNA NC mimic in the HEK-293, U87, SK-N-SH and SH-SY5Y cells. All differences were statistically significant (*p* < 0.001, *p* = 0.032, 0.024 and *p* < 0.001, respectively). However, the hsa-miR-3177-5p inhibitor significantly increased protein expression of the −17 bp to +443 bp sequence only in HEK-293 cells (*p* = 0.036). More importantly, comparing the pmirGLO-HTR1A (−17–+443) mut1 (delete hsa-miR-3177-5p recognition site-tgtacaca) + hsa-miR-3177-5p mimic and pmirGLO-HTR1A (−17–+443) mut1 + miRNA NC mimic, the relative chemiluminescence intensity was not significantly different in the four cell lines (*p* = 0.2, 0.722, 0.715 and 0.176, respectively).

**Figure 5:**
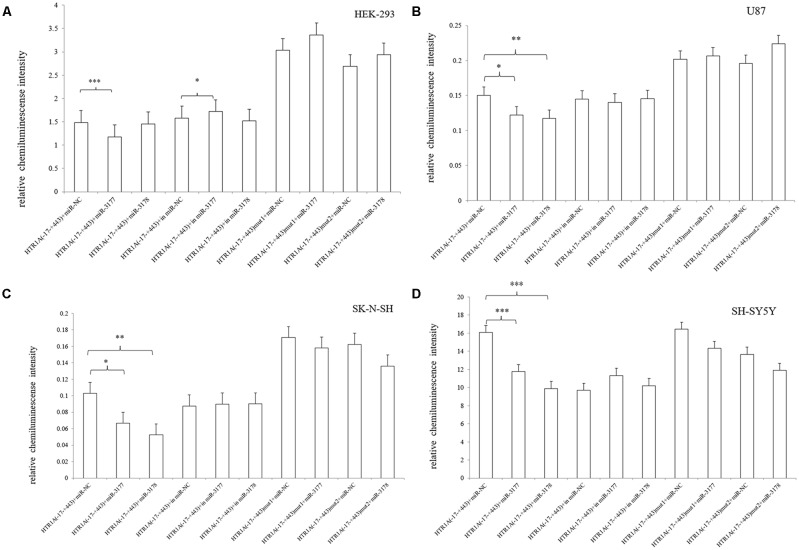
Effects of hsa-miR-3177-5p and hsa-miR-3178 on HTR1A gene expression **(A–D)**. The relative chemiluminescence intensity of the pmirGLO-HTR1A (−17–+443) + hsa-miR-3177-5p mimic was lower than that of the pmirGLO-HTR1A (−17–+443) + miRNA NC mimic in the HEK-293, U87, SK-N-SH and SH-SY5Y cells. However, hsa-miR-3178 inhibited protein expression of the −17 bp to +443 bp sequence only in the U87, SK-N-SH and SH-SY5Y nervous system cell lines. Importantly, when the miRNAs recognition sites were deleted, inhibition by hsa-miR-3177-5p and hsa-miR-3178 disappeared. *0.02 ≤ *p* < 0.05, **0.001 ≤ *p* < 0.02, ***p < 0.001.

It was remarkable that hsa-miR-3178 inhibited protein expression of the −17 bp to +443 bp sequence only in the U87, SK-N-SH and SH-SY5Y nervous system cell lines. The relative chemiluminescence intensities of pmirGLO-HTR1A (−17–+443) + hsa-miR-3178 mimic and pmirGLO-HTR1A (−17–+443) + miRNA NC mimic were compared (*p* = 0.012, 0.002 and *p* < 0.001, respectively). Moreover, this significant inhibition disappeared between the pmirGLO-HTR1A (−17–443) mut2 (delete the hsa-miR-3178 recognition sequence-cgcgccca) + hsa-miR-3178 mimic and pmirGLO-HTR1A (−17–+443) mut2 + miRNA NC mimic (*p* = 0.076, 0.447 and 0.261; Figure 5).

### hsa-miR-3177-5p and hsa-miR-3178 Showed Significant Regulation of the Endogenous 5-HT1A Receptor

**Figure 6:**
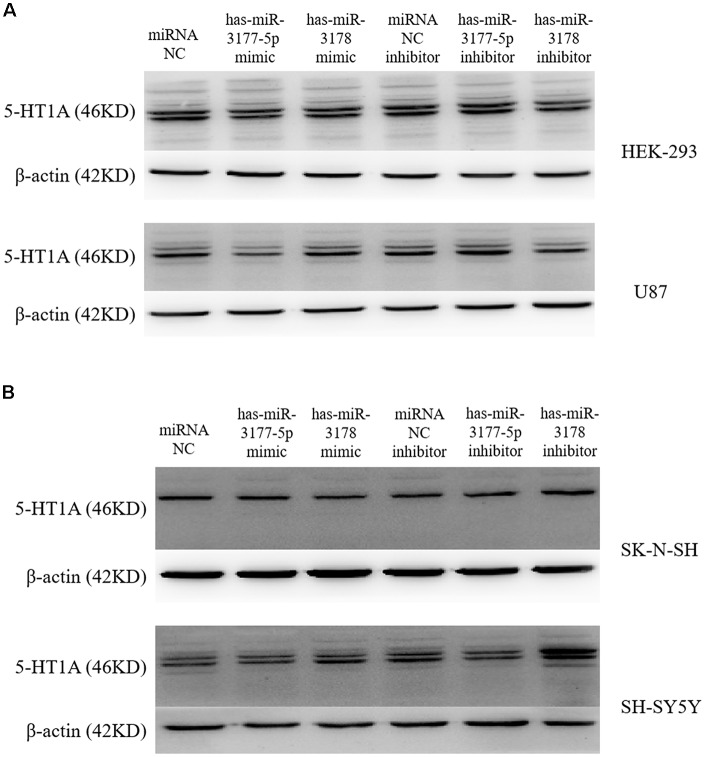
Inhibitory effect of hsa-miR-3177-5p and hsa-miR-3178 on endogenous 5-HT1A receptors **(A)**. Western blot analysis showed that hsa-miR-3177-5p significantly inhibited endogenous 5-HT1A receptor expression in HEK-293 and U87 cells. Only in the U87 cells, the hsa-miR-3177-5p inhibitor slightly increased 5-HT1A receptor expression without reaching statistical significance. **(B)** Results showed that the hsa-miR-3178 inhibitor significantly increased endogenous 5-HT1A receptor expression in SK-N-SH and SH-SY5Y cells. However, in SK-N-SH cells, hsa-miR-3177-5p and hsa-miR-3178 mimics might down-regulate 5-HT1A expression. Beta-actin is shown as the internal reference protein.

**Figure 7:**
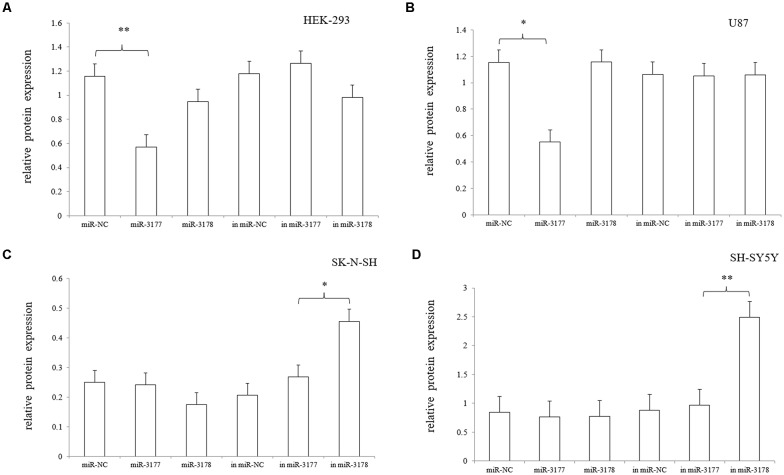
Comparison of the gray values **(A–D)**. The effects of the hsa-miR-3177-5p mimic and hsa-miR-3178 inhibitor on endogenous 5-HT1A receptor reached statistical significance. Gray values for each sample were normalized using β-actin (relative protein expression) and are expressed as the mean ± SD. Student’s *t*-test was used for differential comparison. *0.02 ≤ *p* < 0.05, **0.001 ≤ *p* < 0.02.

Independent transfection of the hsa-miR-3177-5p mimic/inhibitor, hsa-miR-3178 mimic/inhibitor and miR-NC mimic/inhibitor demonstrated that the hsa-miR-3177-5p mimic significantly inhibited expression of the endogenous 5-HT1A receptor in the HEK-293 and U87 cell lines compared with the miR-NC mimic (*p* = 0.002 and 0.037). In the SK-N-SH and SH-SY5Y cell lines, the hsa-miR-3178 inhibitor significantly increased expression of the endogenous 5-HT1A receptor, which was significantly different compared with the miR-NC inhibitor (*p* = 0.037 and 0.015, respectively; Figures 6, 7).

## Discussion

This experiment revealed an important functional sequence located at +375 bp to +443 bp in the 3′-UTR region of the HTR1A gene. Controlling 5-HT1A receptor expression might depend on hsa-miR-3177-5p or hsa-miR-3178. Protein expression of the target fragment −17 bp to +443 bp, which contains the complete HTR1A gene 3′-UTR region, was significantly decreased in the HEK-293, U87 and SK-N-SH cell lines. However, there was no significant change in mRNA expression. We found that the down-regulation of the HTR1A gene 3′-UTR sequence was firmly restored in the SK-N-SH and U87 cell lines that knocked down the Dicer enzyme. In the HEK-293 cells with reduced expression of the Dicer, the inhibition of the gene expression by the 3′-UTR sequence also showed a slight insignificance. The results fully demonstrated that the inhibitory function of the 3′-UTR sequence might be exerted by the Dicer-mediated miRNAs. Most miRNAs bind to the 3′-UTR region of the target gene, which may affect the stability of mRNA and lead to degradation. However, the most important role for miRNAs is to rely on the “seed” region to recognize mRNAs and inhibit translation initiation or protein expression (Carthew, 2006). The results demonstrate that the 3′-UTR sequence of the HTR1A gene might regulate 5-HT1A receptor expression through miRNAs. Previous studies have shown that miRNAs regulate expression of various G-protein coupled receptors, such as β-adrenergic receptor (Wang et al., 2011), adenylate A2A receptor (Tian et al., 2016), estrogen receptor (Adams et al., 2007), dopamine receptor (Huang and Li, 2009) and serotonin receptor (Jensen et al., 2009). Through sequence truncation experiments, we found that only the +375 bp to +443 bp sequence significantly reduced protein expression. MiRNAs are more inclined to recognize the adding A signal-AATAAA region in mRNAs (Sheets et al., 1990) and this fragment (+375 bp to +443 bp) just contains the poly A tail and the adding A signal. TargetScanHuman 7.2 software predicted that hsa-miR-3177-5p would bind to the +377 bp to +384 bp fragment and that hsa-miR-3178 would recognize the +392 bp to +399 bp sequence. Co-transfecting miRNA mimics or inhibitors with the functional fragments confirmed that hsa-miR-3177-5p and hsa-miR-3178 could significantly down-regulate the relative chemiluminescence intensity of the −17 bp to +443 bp sequence. More importantly, the inhibitory effect of hsa-miR-3177-5p on endogenous 5-HT1A receptor expression in HEK-293 and U87 cells remained significant. By competitively binding to endogenous hsa-miR-3178, the hsa-miR-3178 inhibitor significantly increased 5-HT1A receptor expression in SK-N-SH and SH-SY5Y cells. When miRNA recognition sequences were deleted, inhibition of HTR1A gene expression by hsa-miR-3177-5p and hsa-miR-3178 disappeared, which proved that hsa-miR-3177-5p and hsa-miR-3178 exert their functions through combinations with the sequences tgtacaca and cgcgccca.

The related studies have shown that the disorders of the serotonin system, including abnormalities in the 5-HT1A receptor expression and the serotonin neurotransmitter level, lead to the development of neuropsychiatric diseases, such as major depression, anxiety, epilepsy and schizophrenia. Therefore, functional studies of the HTR1A gene regulatory region have attracted much attention. Several transcription factors—Deaf-1 (Huggenvik et al., 1998), Hes-1 (Jacobsen et al., 2008) or Freud-1, 2 (Hadjighassem et al., 2009; Szewczyk et al., 2010), which are acting on the 5′-regulatory region, have been reported. The polymorphism rs6295 (−1019G/C) G allele resulted in a decrease in the HTR1A mRNA level in the hippocampus of patients with epilepsy by altering the binding intimacy of the c-Jun transcription factor (Pernhorst et al., 2013). Meanwhile, rs878567 (*287T/C/G) in the 3′-UTR area also showed the significant interaction with the depression and the life stress (Mekli et al., 2011). This study confirmed that the hsa-miR-3177-5p and hsa-miR-3178, which bound to the 3′-UTR region of the HTR1A gene, significantly inhibited the expression of the HTR1A gene and the 5-HT1A receptor. We hypothesized that the abnormal expression of the miRs might be existed in the brain region or blood of the patient with depression or schizophrenia, resulting in the change of the 5-HT1A receptor expression.

Expression of hsa-miR-3177 was shown to be increased in the skin tissue of patients with myeloid granulomatous tumor stage (tMF), revealing that hsa-miR-3177 might be a new therapeutic treatment for the disease (Papadavid et al., 2016). The present study demonstrated that hsa-miR-3177 not only regulated tumor diseases, but might also play a crucial role in the nervous system. MiRNA real-time PCR showed that hsa-miR-3177-5p was strongly expression in four cell lines (*C*_T_ values range from 26 to 28), which might indicate that miRNA has a wide regulatory effect on the target gene. However, hsa-miR-3177-5p displayed only weak inhibitory effects on endogenous 5-HT1A receptor expression in SK-N-SH and SH-SY5Y cells, which did not reach statistical difference. A large number of miRNAs are present in cells. Each miRNA can either cooperate or compete with others to constitute a “trimmer” in the miRNA network (Zhou et al., 2016). In fact, every miRNA has a subtle regulatory effect on the target gene, resulting in variations in miRNA content that may not sufficient to cause dramatic changes in target gene expression. Studies have also shown that efficient binding between miRNA and mRNA requires co-factors (Jensen et al., 2009). SK-N-SH and SH-SY5Y cells might lack additional factors that could assist the recognition of hsa-miR-3177-5p. We found that hsa-miR-3178 only significantly inhibited 5-HT1A expression in the nervous system cell lines U87, SK-N-SH and SH-SY5Y. However, the endogenous 5-HT1A receptor in HEK-293 cells was not sensitive to inhibition by hsa-miR-3178. These results suggest that hsa-miR-3178 might be specific for regulation in the nervous system. Changes in hsa-miR-3178 content may directly lead to 5-HT1A receptor-mediated neurological diseases. Relevant reports identified a significant increase in hsa-miR-3178 in surgically resected specimens from breast cancer patients (Zhou et al., 2018), and hsa-miR-3178 was associated with drug resistance in breast cancer treatments (Chen et al., 2016). The presence of the helicobacter pylori carcinogenic factor Tip-a inhibited miR-3178 expression, leading to inflammation of the stomach and carcinogenesis (Zou et al., 2017). In addition, research on hsa-miR-3178 has mainly involved gastric cancer metastasis (Yang et al., 2014), gastric stromal tumor (Xiao et al., 2015), hepatocellular carcinoma (Li et al., 2015) and the immune system (Das et al., 2013). Interestingly, abnormal regulation of the serotonin system and 5-HT1A receptor has been implicated in gastrointestinal irritation (Sun et al., 2016) and changes in the immune system and inflammation (Hernández-Torres et al., 2018). Therefore, it was further identified that hsa-miR-3178 likely has an important regulatory function in affecting 5-HT1A receptor expression, which might cause susceptibility to neurological diseases.

Current studies on the effects of miRNAs on the serotonin system have confirmed that miR-96, miR-30c and miR-103/107 inhibited expression of the HTR1B, HTR2A and HTR4 receptor, respectively (Sánchez-Mora et al., 2013; Wohlfarth et al., 2017). SLC6A4 gene expression was shown to be regulated by a variety of miRNAs, such as miR-16, miR-135, miR-545 and miR-590-3p (Iurescia et al., 2017). Research on miRNAs regulating the HTR1A gene revealed that decreased miR-135a expression was associated with an increase in the 5-HT1A receptor in the prefrontal cortex when male rats were exposed to stressful conditions. An increase in miR-16 was also found in the hippocampus of the rat brain. This suggests that regulation of miRNAs is critical for the expression of the serotonin system and the 5-HT1A receptor. The two miRNAs we validated in the present study, hsa-miR-3177-5p and hsa-miR-3178, increase the likelihood that the miRNA system regulates 5-HT1A receptor expression.

Although we used four cell lines to confirm that hsa-miR-3177-5p and hsa-miR-3178 significantly inhibited 5-HT1A receptor expression, there were still several shortcomings. In the experiment, the mimics/inhibitors of hsa-miR-3177-5p and hsa-miR-3178 were mature miRNAs, but studies have demonstrated that expression of miRNA precursors can directly affect the role of mature miRNAs and that the ratio of the two in cells or tissues may also influence the effects of miRNAs on the target genes (Chak et al., 2016). Therefore, subsequent studies should incorporate miRNA precursors. Given that miRNA content in the blood circulation may also represent expression in the brain tissue, it could be possible to analyze differences of miRNA expression in fresh blood between patients with neuropsychiatric diseases and healthy subjects in future research. Although a variety of cell lines were evaluated in this study, perhaps *in vivo* experiments would provide more comprehensive and reliable results.

In summary, in HEK-293, U87, SK-N-SH and SH-SY5Y cell lines, the complete 3′-UTR region of the HTR1A gene was shown to have a significant inhibitory effect on protein expressions, but there was no significant change in mRNA levels. The fragment located at +375 bp to +443 bp down-regulated 5-HT1A receptor expression. hsa-miR-3177-5p significantly reduced the number of endogenous 5-HT1A receptors in HEK-293 and U87 cells, while 5-HT1A receptor expression was significantly increased by a hsa-miR-3178 inhibitor in SK-N-SH and SH-SY5Y cells. Importantly, we also demonstrated that hsa-miR-3177-5p and hsa-miR-3178 exerted their functions through combinations with the sequences tgtacaca and cgcgccca. These data show that hsa-miR-3177-5p and hsa-miR-3178 have important regulatory effects on expression of the HTR1A gene and 5-HT1A receptor and may be directly involved in the development of mental illness. In the future studies, the expression of hsa-miR-3177-5p and hsa-miR-3178 could be further verified using the brain tissue or blood of patients with mental illness. In order to ensure the normal function of miRNA and the precise regulation of target genes, the studies on the regulation of the miRNA synthesis should also be concerned.

## Author Contributions

XW conducted the experimental operation, data summary and the preparation of this article. XW, YL, XX and FX researched the study program. BW, JY and MD gave the experimental design ideas, experimental guidance and financial support. All authors read and approved the final manuscript.

## Conflict of Interest Statement

The authors declare that the research was conducted in the absence of any commercial or financial relationships that could be construed as a potential conflict of interest.

## Footnotes

^1^www.targetscan.org

^2^www.mirbase.org
