# Supplementary material for: hsa-miR-3177-5p and hsa-miR-3178 Inhibit 5-HT1A Expression by Binding the 3′-UTR Region in vitro
Source: Front Mol Neurosci. 2019 Jan 31;12:13. doi: 10.3389/fnmol.2019.00013 (PMC6365703; doi:10.3389/fnmol.2019.00013)
Supplement: Supplementary file 1 [file Data_Sheet_1.doc]

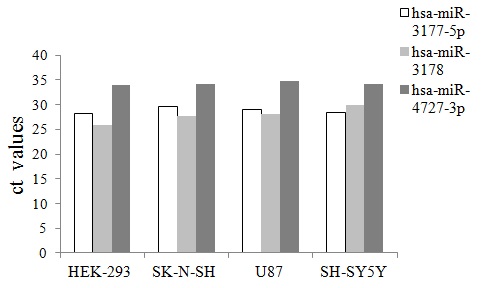


**Figure S1 Endogenous expression of the miRNAs in the four cell lines.** We used miRNA real time PCR to detect the expression of the endogenous target miRNAs in cells. Due to the low expression of hsa-miR-4727-3p, we finally investigated the effects of the hsa-miR-3177-5p and hsa-miR-3178 on the gene expression.
